# Hemorrhage During Induction Chemotherapy in Neuroblastoma: Additional Risk Factors in High-Risk Patients

**DOI:** 10.3389/fped.2021.761896

**Published:** 2021-11-16

**Authors:** Valerio Voglino, Giorgio Persano, Alessandro Crocoli, Aurora Castellano, Annalisa Serra, Ugo Giordano, Gian Luigi Natali, Pier Luigi Di Paolo, Cristina Martucci, Alessandra Stracuzzi, Alessandro Inserra

**Affiliations:** ^1^Surgical Oncology—General and Thoracic Surgery Unit, Department of Surgery, Bambino Gesù Children's Hospital IRCCS, Rome, Italy; ^2^Onco-Hematology Unit, Department of Onco-Hematology and Gene Therapy, Bambino Gesù Children's Hospital IRCCS, Rome, Italy; ^3^Sport and Hypertension Medicine Unit, Department of Cardiac Surgery, Cardiology, Heart and Lung Transplant, Bambino Gesù Children's Hospital IRCCS, Rome, Italy; ^4^Radiology Unit, Department of Diagnostic Imaging, Bambino Gesù Children's Hospital IRCCS, Rome, Italy; ^5^Pathology Unit, Department of Laboratories, Bambino Gesù Children's Hospital IRCCS, Rome, Italy

**Keywords:** high-risk neuroblastoma, hemorrhagic complications, hemothorax, hemoperitoneum, chemotherapy complications

## Abstract

**Background:** Neuroblastoma is the most common solid extracranial tumor in children. Patients affected by neuroblastoma are stratified into low, intermediate, and high risk in terms of event-free and overall survival. Some high-risk patients have an additional risk of acute hemorrhagic complications during induction chemotherapy.

**Aim:** To find easily and rapidly assessed parameters that help clinicians identify those patients affected by high-risk neuroblastoma who have an additional risk of hemorrhagic complications.

**Methods:** The clinical notes of patients diagnosed with high-risk neuroblastoma from January 2013 until February 2021 were retrospectively reviewed. Clinical, demographic and laboratory data, biological characteristics of the tumor, and information about treatment and hospital stay were identified.

**Results:** In the examined period, 44 patients were diagnosed with high-risk neuroblastoma. Four of these patients had hemorrhagic complications within 2–7 days after the initiation of induction chemotherapy; two patients had hemothorax, one patient had hemoperitoneum and one patient had hemothorax and hemoperitoneum. The patient with isolated hemoperitoneum was treated with blood components transfusions, clotting factors and colloids infusions; the three patients with hemothorax underwent thoracostomy tube placement and respiratory support. At initial presentation, patients who suffered from hemorrhagic complications had a higher degree of hypertension (stage 2, *p* = 0.0003), higher levels of LDH (median 3,745 U/L, *p* = 0.009) and lower levels of hemoglobin (mean 7.6 gr/dl, *p* = 0.0007) compared to other high-risk patients.

**Conclusions:** A subgroup of “additional” high-risk patients can be identified within the high-risk neuroblastoma patients based on mean arterial pressure, LDH levels and hemoglobin levels at presentation. Further studies to define cut-off values and optimal management strategies for these patients are needed.

## Introduction

Neuroblastoma is the most common solid extracranial tumor in childhood worldwide, accounting for 8–10% of all cancer cases in children (1); it arises from the neural crest cells of the developing sympathetic system, typically resulting in adrenal or paravertebral tumors (2). Staging and pretreatment risk stratification of neuroblastoma are based on the International Neuroblastoma Risk Group (INRG) staging and classification system; patients are divided into low, intermediate and high risk in terms of Event-Free Survival (EFS) and Overall Survival (OS) (3, 4).

Clinical presentation of neuroblastoma varies widely, ranging from asymptomatic patients to symptoms related to local compression of adjacent structures and to catecholamines or vasoactive intestinal peptide (VIP) secretion, such as hypertension and intractable diarrhea, systemic non-specific symptoms, such as fever and weight loss, or cytopenia related to bone marrow metastases (5, 6).

On rare instances, children with neuroblastoma may present with acute hemorrhage such as hemothorax and hemoperitoneum (7–9). Such cases pose a great challenge for the caring clinicians.

The purpose of this study is to identify and describe a specific subset of high-risk patients who have additional risk of developing hemorrhagic complications, in order to find rapidly and easily assessed parameters that can help clinicians predict these complications and optimize their treatment.

## Patients and Methods

All the patients diagnosed with high-risk neuroblastoma at Bambino Gesù Children's Hospital from January 2013 until February 2021 were included in the study. Risk stratification was performed according to the criteria of the International Neuroblastoma Risk Group (INRG) Classification System (3); all the patients were evaluated and treated according to the High Risk Neuroblastoma Study 1.8 of SIOP-Europe (SIOPEN) (10). Patients who were referred from other institutions after the diagnosis had already been established were excluded from the present study.

At presentation, all the patients underwent full clinical assessment, serial measurement of arterial pressure, complete blood count, lactate dehydrogenase (LDH) and uric acid serum levels, urinary catecholamine metabolites, i.e., vanilmandelic acid (VMA) and homovanillic acid (HVA), coagulation tests, hepatic and renal function tests.

All the patients underwent total-body contrast-enhanced computed tomography (CT) and meta-iodobenzylguanidine (MIBG) scintigraphy.

Diagnosis was confirmed by histology performed on core needle biopsy and amplification of N-MYC on tumor specimens was determined for every patient. All the patients underwent bone marrow biopsy as part of initial work-up.

Patients were divided in two groups; patients who developed hemorrhagic complications during induction chemotherapy were categorized in group A, while patients who did not develop such complications were categorized in group B.

In order to differentiate anemia secondary to chemotherapy-induced bone marrow aplasia form anemia secondary to blood loss, hemorrhagic complications were defined by the concurrent presence of the following three criteria: (1) anemia (i.e., hemoglobin levels below 8.0 gr/dL) that persisted after the transfusion of 10 mL/kg of packed red cells, (2) the presence of respiratory distress or abdominal pain or distension, (3) radiological evidence of pleural effusion or free abdominal fluid.

The following variables were analyzed: clinical features, laboratory findings, radiologic assessment, histology/biology (see detailed description below).

Statistical analyses were performed using Prism 9.0.0.121 (GraphPad Software, Inc., San Diego, CA).

Categorical variables were analyzed using Fisher's test. Continuous variables were tested for normal distribution using D'Agostino-Pearson test: variables with normal distribution were analyzed using Student's *t*-test, while variables without normal distribution were analyzed with Mann-Whitney test.

Variables that resulted statistically significant on univariate analysis were subsequently tested on multivariate logistic regression; the outcome (dependent) variable was the occurrence of hemorrhage.

A value of *p* < 0.05 was considered statistically significant for each analysis.

### Clinical Features

Age at diagnosis: median age at diagnosis in months was calculated separately in the two groups and data have been compared using Mann-Whitney test.

Time from onset of symptoms to diagnosis: time in weeks from onset of symptoms to diagnosis was recorded for each patient from the history reported in the clinical notes. Median time and range were calculated in each group and data were compared using Mann-Whitney test.

Systemic symptoms: the presence of fever >37.5°C, weight loss or asthenia was recorded for each patient in the two groups. Data were compared using Fisher's test.

Arterial pressure: as per institutional protocol, arterial pressure measurements were performed upon admission and every 8 h for each patient. Mean values for the first 4 days from admission were calculated for each patient. Patients were defined as having normal blood pressure, stage 1 hypertension or stage 2 hypertension according to the “Clinical Practice Guideline for Screening and Management of High Blood Pressure in Children and Adolescents” published in 2017 (11). Patients were grouped according to the presence of stage 2 hypertension vs. stage 1 or no hypertension. Data were compared using Fisher's test.

### Laboratory Findings

Hemoglobin levels: full blood count was performed in every patient upon admission. Mean hemoglobin level and standard deviation (SD) have been calculated separately in the two groups and data have been compared using Student's *t*-test.

LDH levels: LDH serum levels were measured in every patient upon admission. Median levels and range have been calculated separately in the two groups and data have been compared using Mann-Whitney test.

Urinary VMA and HVA levels: VMA and HVA urinary levels were measured in every patient upon admission. Median levels and range for the two metabolites have been calculated separately in the two groups and data have been compared using Mann-Whitney test.

### Radiologic Assessment

Maximum diameter of the primary tumor: maximum diameter of the primary tumor was measured for each patient on initial CT images by the radiologist who performed the investigation and subsequently revised by GLN and PDP. Mean diameter and standard deviation (SD) have been calculated separately in the two groups and data have been compared using Student's *t*-test.

Vascular and total Image-Defined Risk Factors (IDRF): the presence and number of both vascular and total IDRF was assessed for each patient on initial CT images by the radiologist who performed the investigation and subsequently revised by GLN and PDP. IDRF were defined according to the International Neuroblastoma Risk Group (INRG) Staging System (4). Mean number and standard deviation (SD) have been calculated separately in the two groups and data have been compared using Student's *t*-test.

Stage: patients were staged by CT scan and MIBG scintigraphy according to the International Neuroblastoma Risk Group (INRG) Staging System (4). Stage distributions in the two groups were compared using Fisher's test.

### Histology/Biology

Bone marrow infiltration: bone marrow biopsy was performed at initial presentation in every patient. Patients were grouped according to the presence vs. absence of neuroblastoma infiltrates in the bone marrow. Data were compared using Fisher's test.

N-MYC amplification: amplification of N-MYC on biopsy specimens was determined for every patient. Patients were grouped according to the presence vs. absence of N-MYC amplification. Data were compared using Fisher's test.

## Results

In the examined period, 44 patients were diagnosed with High-risk neuroblastoma at our institution and were included in the present study. None of the patient had pre-existing comorbidities and coagulation tests, hepatic and renal function tests did not reveal any abnormality in any patient.

All the patients had avid uptake on MIBG scan.

All the patients received induction chemotherapy according to the Rapid COJEC schedule of the High Risk Neuroblastoma Study 1.8 of SIOP-Europe (SIOPEN) (10).

**Figure 1:**
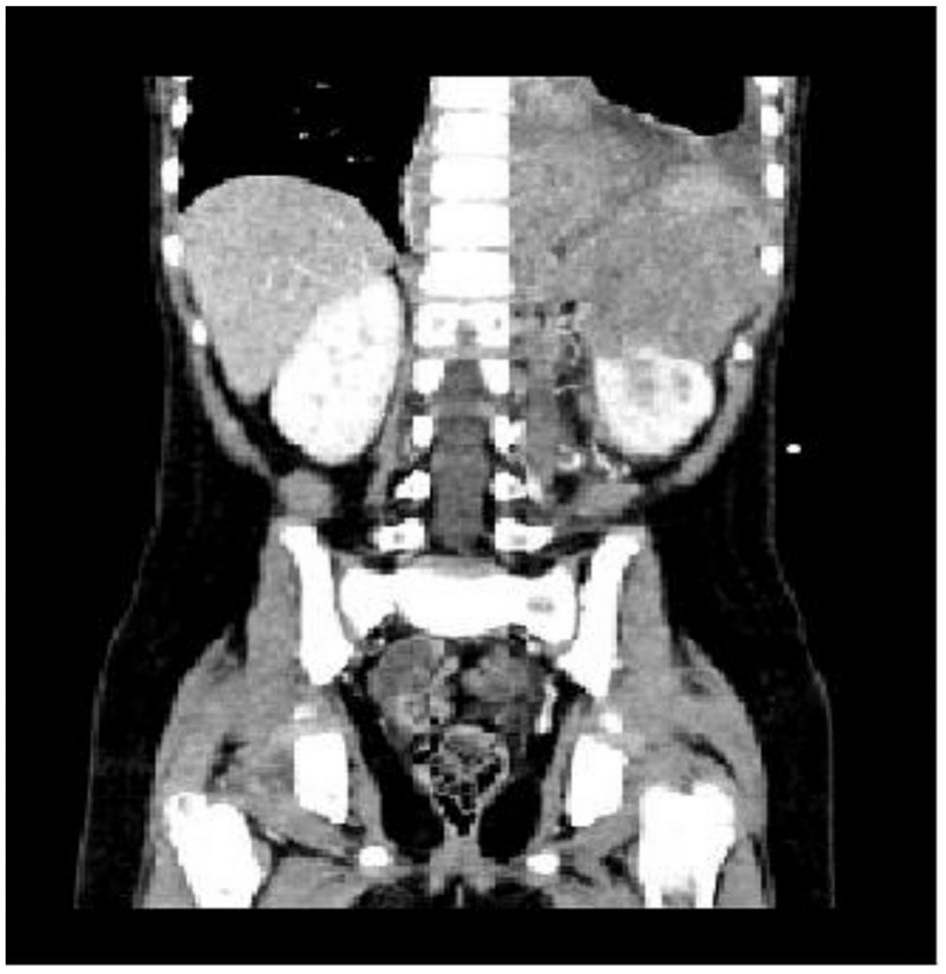
Left retroperitoneal neuroblastoma with posterior mediastinum extension.

Four patients (9%) developed hemorrhagic complications within 2–7 days (mean 3.25 days) after the administration of the first course of chemotherapy and were categorized in group A; two patients had hemothorax, one patient had hemoperitoneum and one patient had hemothorax and hemoperitoneum. All these patients had primary left retroperitoneal tumors, one patient also had extension of neoplastic tissue in the posterior mediastinum (Figure 1) and all of them presented with encasement of the aorta, the celiac tripod, the superior mesenteric artery and the left renal pedicle. Two patients had stage L2 disease, and two patients had stage M disease and no one of them had evidence of active bleeding on the initial staging CT scan. The clinical features of these patients at diagnosis and at the onset of hemorrhage are summarized in Tables 1, 2, respectively.

**Table 1:** Patients' characteristics at initial presentation (group A).

**Patient**	**Sex**	**Age (months)**	**Presentation**						**Laboratory**				**Radiology**						**Biology**		**Complications**	
			**Systemic symptoms**	**HR Mean**	***Centile***	**SBP Mean**	***Centile***	**HTN**	**LDH**	**Hb**	**VMA**	**HVA**	**Primary**	**Side**	**IDRF** **Vascular**	**Total**	**Max diam** **(cm)**	**Stage**	**n-MYC**	**Bone** **marrow**	**Hemo** **thorax**	**Hemoperi** **toneum**
1	M	17	Yes	123	75th	121	>95th + 12 mmHg	2	4,561	7.6	16.8	20	Abd	Left	4	4	13.5	M	ampl	Neg	Yes	
2	M	19	Yes	145	95th	125	>95th + 12 mmHg	2	2,834	6.6	9	33	Abd	Left	4	4	14	L2	ampl	Neg	Yes	Yes
3	F	47	Yes	106	75th	123	>95th + 12 mmHg	2	2,928	8.7	24	197	Thor-Abd	Left	4	5	17	M	ampl	Pos	Yes	
4	M	15	Yes	123	75th	126	>95th + 12 mmHg	2	6,677	7.6	6.25	22	Abd	Left	4	4	14	L2	ampl	Neg		Yes

*HR, Heart Rate; SBP, Systolic Blood Pressure (mmHg); HTN, Hypertension (stage); LDH, lactate dehydrogenase (U/L); Hb, hemoglobin (gr/dL); VMA, Vanilmandelic acid (mcg/mg creat); HVA, Homovanillic acid (mcg/mg creat); IDRF, Image-Defined Risk Factors*.

**Table 2:** Patients' characteristics at onset of hemorrhage (group A).

**Patient**	**Onset of hemorrhage days from chemotherapy**	**Complication**		**Hemodynamics**						Lysis marker^*^	
		**Hemothorax**	**Hemoperitoneum**	**HR**			**SBP**			**LDH**	**Variation (x-fold)**
				**Mean**	**Centile**	**% Variation**	**Mean**	**Centile**	**% Variation**		
1	2	Yes		153	99th	24	112	95th + 4 mmHg	−7	21,495	x 4.7
2	2	Yes	Yes	146	95th	0	114	95th + 6 mmHg	−9	11,163	x 3.9
3	7	Yes		132	95th	25	104	90th	−15	12,959	x 4.4
4	2		Yes	140	90th	14	107	95th	−15	20,771	x 3.1

*HR, Heart Rate; SBP, Systolic Blood Pressure (mmHg); LDH, lactate dehydrogenase (U/L)*.

* *Lysis marker value is measured as the highest level of LDH in the seven days following the first course of chemotherapy*.

**Figure 2:**
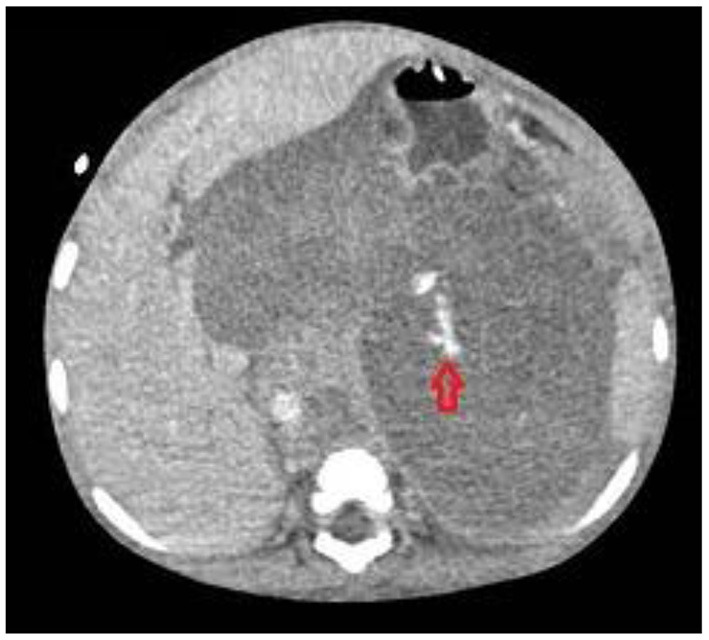
Bleeding from left diaphragmatic artery (CT scan: circle).

**Figure 3:**
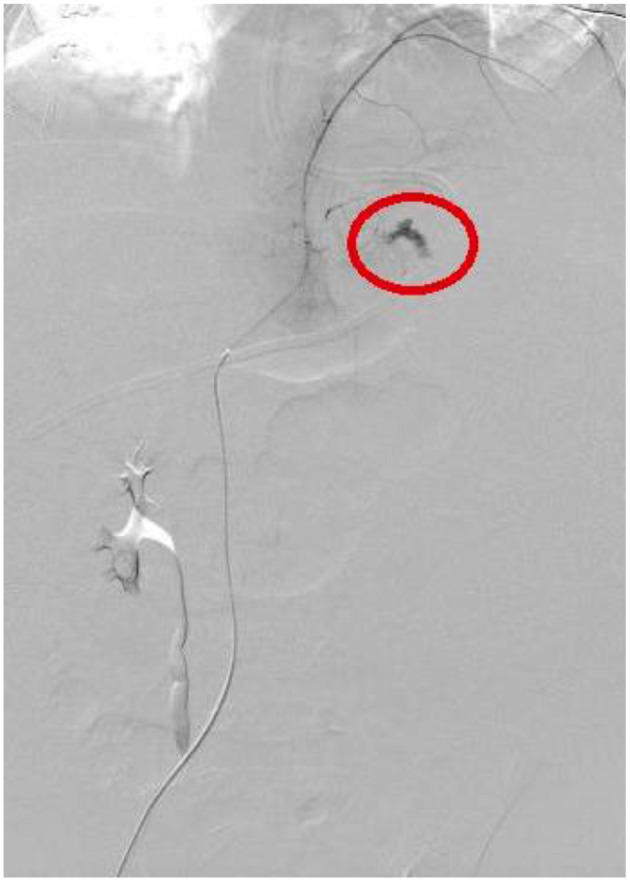
Bleeding from left diaphragmatic artery (angiography: circle).

**Figure 4:**
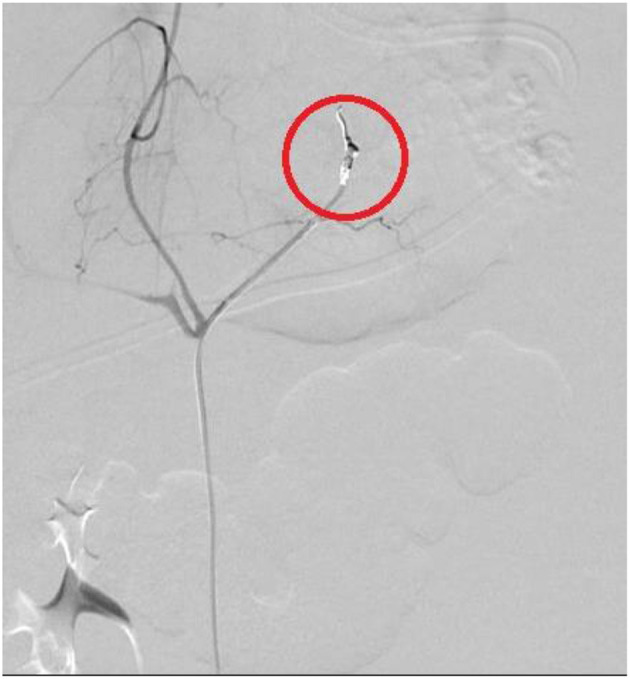
Embolization of left diaphragmatic artery (angiography: circle).

All these four patients received medical treatment consisting in packed red cells and platelets transfusions, plasma or purified vitamin K-dependent clotting factors infusions and albumin infusions. The two patients with isolated hemothorax underwent thoracostomy tube placement. The patient with associated hemothorax and hemoperitoneum had radiological evidence of bleeding from the left diaphragmatic artery (Figures 2, 3); this patient underwent thoracostomy tube placement and angio-embolization of the bleeding vessel (Figure 4). The patient with isolated hemoperitoneum received medical treatment only.

All the patients successfully recovered after the hemorrhagic complications. Table 3 summarizes the treatment for each patient. One patient died of progressive disease 4 months after diagnosis, two patients are currently on first line treatment and one patient is in complete remission with a follow up of 52 months.

**Table 3:** Patients' treatment (group A).

**Patient**	**Complication**		**Medical treatment**							**Operative treatment**		**Respiratory support**	**ICU stay**
	**Hemothorax**	**Hemoperitoneum**	**Amlodipine**	**Carvedilol**	**Albumine**	**Blood prod** **red cells**	**Platelets**	**Clotting fact** **Plasma**	**Purified vit-k factors**	**Chest tube**	**Embolization**		
1	Yes		0.3 mg/Kg/day	0.5 mg/Kg/day	1.0 gr/kg x 6 dd	20 mL/kg		2 units	20 UI/Kg	Yes		HFNC (FiO2 30%; 20 l/min) x 3 days	
2	Yes	Yes	0.3 mg/Kg/day	0.5 mg/Kg/day	1.5 gr/kg x 4 dd	120 mL/kg	10 units	1 unit	45 UI/Kg	Yes	Yes	Mechanical ventilation x 5 days	6 days
3	Yes		0.3 mg/Kg/day	0.4 mg/Kg/day	0.5 gr/Kg x 10 dd	40 mL/kg	4 units			Yes		Mechanical ventilation x 3 days	9 days
4		Yes	0.4 mg/Kg/day	0.6 mg/Kg/day	0.5 gr/Kg x 10 dd	40 mL/kg	8 units		50 UI/Kg				

*HFNC, High Flow Nasal Cannula*.

Forty patients (91%) did not develop hemorrhagic complications and were therefore categorized in group B. Thirty-seven patients (92.5%) had primary retroperitoneal tumor, with mediastinal extension in two cases. Two patients (5%) had primary mediastinal tumors and one patient (2.5%) had primary cervical localization. Four patients (10%) had stage L2 disease and 36 (90%) had stage M disease. None of these patients developed hemorrhagic complications during subsequent courses of chemotherapy.

### Clinical Features

Age at diagnosis: median age for patients in group A was 18 months (range 15–47 months) while mean age in group B was 42 months (range 11–199 months). Patients in group A were significantly younger than patients in group B (*p* = 0.0343; Mann-Whitney).

Time from onset of symptoms to diagnosis: median time was 3 weeks in group A (range 1–4 weeks) and 4 weeks in group B (range 1–22 weeks). The difference between the two groups was not statistically significant (*p* = 0.2741; Mann-Whitney).

Systemic symptoms: all the patients (100%) in group A presented with systemic symptoms while 26 patients (72.5%) in group B had systemic symptoms at diagnosis. There was no statistically significant difference between the two groups (*p* = 0.2897; Fisher's).

Arterial pressure: all the patients in group A (100%) presented with stage 2 hypertension while 3 patients (7.5%) in group B had stage 2 hypertension. Patients in group A had a statistically significant higher severity of hypertension (*p* = 0.0003; Fisher's).

### Laboratory Findings

Hemoglobin levels: mean hemoglobin level was 7.6 gr/dL (SD 0.9 gr/dL; range 6.6–8.7 gr/dL) in group A and 9.9 gr/dL (SD 1.9 gr/dL; range 5.8–14.7 gr/dL) for group B. The difference between the two groups was statistically significant (*p* = 0.0007; Student's *t*-test).

LDH levels: median LDH serum level was 3,745 IU/L (range 2834–6677 IU/L) for group A and 1,089 IU/L (range 366–8,640 IU/L) for group B. Patients in group A had statistically significant higher levels of LDH compared to group B (*p* = 0.009; Mann-Whitney).

Urinary VMA and HVA levels: median VMA level was 12.9 mcg/mg creat (range 6.25–24.0 mcg/mg creat) for group A and 98.4 mcg/mg creat (range 5.0–2657.0 mcg/mg creat) for group B. The difference was statistically significant (*p* = 0.0048; Mann-Whitney).

Median HVA level was 27.5 mcg/mg creat (range 20.0–197.0 mcg/mg creat) for group A and 128.1 mcg/mg creat (range 9.5–2,191.0 mcg/mg creat) for group B. The difference was not statistically significant (*p* = 0.0612, Mann-Whitney).

### Radiologic Assessment

Maximum diameter of the primary tumor: mean value for maximum diameter of the primary tumor was 14.6 cm (SD 1.6 cm; range 13.5–17.0 cm) for group A and 10.9 cm (SD 3.8 cm; range 4.1 – 18.0 cm). The difference between the two groups was not statistically significant (*p* = 0.0625, Student's *t*-test).

Vascular and total Image Defined Risk Factors (IDRF): all the patients in group A had 4 vascular IDRF, while mean number of vascular IDRF in group B was 2.2 (st. dev 1.1; range 0–4). The difference was statistically significant (*p* = 0.0024, Student's *t*-test).

The mean number of total IDRF was 4.2 (SD 0.5; range 4–5) in group A and 3.2 (SD 1.4; range 0–5) in group B. The difference between the two groups was not statistically significant (p = 0.0752, Student's *t*-test).

Stage: in group A 2 patients had stage L2 disease and 2 patients had stage M disease 50–50%). In group B 4 patients had stage L2 disease and 36 had stage M disease (10–90%). The difference was not statistically significant (*p* = 0.0834, Fisher's).

### Histology/Biology

Bone marrow infiltration: one patient (25%) in group A had bone marrow infiltrates while 34 patients (85%) in group B had positive bone marrow biopsy. Patients with hemorrhagic complications had a significantly lower incidence of bone marrow metastases (*p* = 0.0226, Fisher's).

N-MYC amplification: all the patients (100%) in group A had amplification of N-MYC while 23 patients (57.5%) in group B had N-MYC amplification. Such difference was not statistically significant (*p* = 0.1468, Fisher's).

The variables that were associated with bleeding on univariate analysis (i.e., age, stage 2 hypertension, hemoglobin levels, LDH levels, urinary VMA levels, vascular IDRF, bone marrow infiltration) were subsequently tested for multivariate logistic regression; the model resulted in a perfect separation.

Results are summarized in Table 4.

**Table 4:** Comparison and statistics.

	**Group A**		**Group B**		***p*** **-value**	
**Number of patients**	4		40			
**Clinical presentation**						
Age (months: median + range)	18	15–47	42	11–199	0.0343	*Significant*
Time from onset to diagnosis (weeks: median + range)	3	1–4	4	1–22	0.2741	
Systemic symptoms (n. of pts + percentage)	4	100%	26	72.50%	0.2897	
**Arterial pressure (n. of pts** **+** **percentage)**						
Hypertension stage 2	4	100%	3	7.50%	0.0003	*Significant*
Hypertension stage 1	0	0%	7	17.50%		
Normal arterial pressure	0	0%	30	75%		
**Laboratory findings**						
Hb (gr/dL: mean + standard deviation)	7.6	0.9	9.9	1.9	0.0007	*Significant*
LDH (IU/L: median + range)	3,745	2,834–6,677	1,864	366–8,640	0.009	*Significant*
VMA (mcg/mg creat: median + range)	12.9	6.25–24.0	98.4	5.0–2,657.0	0.0048	*Significant*
HVA (mcg/mg creat: median + range)	27.5	20.0–197.0	128.1	9.5–2,191.0	0.0612	
**Radiology**						
Maximum diameter (cm: mean + standard deviation)	14.6	1.6	10.9	3.8	0.0625	
**IDRF (number** **+** **standard deviation)**						
Vascular	4	0	2.2	1.1	0.0024	*Significant*
Total	4.2	0.5	3.2	1.4	0.0752	
Stage (L2 vs. M)	2	2	4	36	0.0834	
**Istology/biology**						
Bone marrow infiltration (n. of pts + percentage)	1	25%	34	85%	0.0226	*Significant*
N-MYC amplification (n. of pts + percentage)	4	100%	23	57.50%	0.1468	

*LDH, lactate dehydrogenase; Hb, hemoglobin; VMA, Vanilmandelic acid; HVA, Homovanillic acid; IDRF, Image-Defined Risk Factors*.

## Discussion

Hemorrhage is an uncommon, life-threatening event in patients affected by neuroblastoma (7, 12, 13). In a recent paper, Qin et al. reported 47 neuroblastoma patients with hemorrhage, either secondary to spontaneous tumor rupture or after chemotherapy or biopsy, on a total population of ~1,800 patients, with an incidence of approximately 2.6% and poor outcome; treatment was withdrawn in 17 of these patients, while other 5 patients died as an immediate consequence of this complication (9).

The mechanism underlying spontaneous hemorrhage in neuroblastoma has been debated; in neonates, an adrenal mass could be crushed between the liver and the spine during delivery, causing tumor rupture and subsequent hemorrhage (14), while in older children the presence of neuroblastoma could predispose to adrenal hemorrhage following minor trauma (15).

Most authors report massive hemorrhage in patients younger than 18 months affected by high risk neuroblastoma with N-MYC amplification (7, 8, 13, 15–17). In their large case series, Qin et al. found two independent risk factors on multivariate analysis, i.e., the presence of N-MYC amplification and high tumor bulk, measured as maximum diameter of the primary mass. These authors also reported younger mean age (29 months vs. 43 months) and higher LDH values (3148.5 U/L vs. 723 U/L) in patients with hemorrhage secondary to tumor rupture compared to other neuroblastoma patients (9).

In the present case series, 4 out of 44 (9%) patients experienced hemorrhage, a proportion that is higher than previous reports (9). All the patients in group A and the majority of patients in group B had N-MYC amplification, without statistically significant difference between the two groups. Both observations are expected due to the selection of high risk patients only in the study.

The presence of systemic symptoms (i.e., fever, weight loss, asthenia) was similar in the two groups. Systemic symptoms are frequently associated with metastatic neuroblastoma (6, 18); a high prevalence of systemic symptoms is therefore anticipated in a population of high risk neuroblastoma patients.

Patients who experienced hemorrhage were significantly younger than other high risk patients; this observation is consistent with previously published data (9).

Patients in group A presented with a higher severity of hypertension compared to group B, i.e., stage 2 according to the clinical practice guidelines of the American Academy of Pediatrics (11). Hypertension is classified as a life-threatening symptom that warrants chemotherapy in the European Low and Intermediate Risk Neuroblastoma Protocol (19), but the severity of hypertension is not part of the risk stratification algorithm and is not reported in previous literature as a risk factor for hemorrhage.

Hypertension in neuroblastoma patients has been associated with catecholamine release, although a linear correlation between the severity of hypertension and urinary catecholamine levels has not been demonstrated (20, 21). In the present case series, patients with hemorrhagic complications had lower levels of urinary VMA compared to patients without hemorrhage. Such observation is consistent with the study by Qin et al. who report lower urinary VMA and HVA levels in patients who experience neuroblastoma rupture. Streger et al. found low levels of urinary VMA in patients with N-MYC amplification and high levels of urinary dopamine in higher stage neuroblastoma (22).

Patients in group A presented with a high mean number of vascular IDRF compared to group B; vascular encasement, especially of the renal artery (20), may contribute to the development of hypertension and also predispose patients to vascular erosion and bleeding.

Patients who had hemorrhagic complications presented with lower hemoglobin levels at presentation compared to patients without hemorrhage despite a lower incidence of bone marrow metastases. Neuroblastoma can alter the microenvironment of bone marrow irrespective of neoplastic cell invasion, causing downregulation of genes involved in cell adhesion, and in erythrocyte, myeloid, and platelet differentiation pathways (23). Neuroblastoma can impair erythropoiesis by selectively disrupting the late stage of erythrocytes' maturation independently of the physical presence of neuroblastoma cells in the bone marrow, thus reducing hemoglobin levels in peripheral blood (24). All the patients in our study were affected by high risk neuroblastoma and therefore neuroblastoma-induced impaired erythropoiesis should have theoretically affected all the patients to a similar extent; patients who are prone to develop hemorrhagic complications may be more susceptible to the mechanism that inhibits erythropoiesis in neuroblastoma patients. Another explanation for lower hemoglobin levels could be related to slow, chronic intratumoral bleeding secondary to vascular erosion in patients who then develops frank hemorrhage; the extent of vascular encasement in these patients might support the second explanation. Large, multicentric series are necessary to clarify this issue.

Patients in group A had significantly higher LDH levels at diagnosis compared to group B; such data are consistent with the work by Qin et al. and other published case reports (8, 9, 17). High serum LDH levels at diagnosis are associated with poorer outcome in terms of event-free and overall survival in high-risk neuroblastoma (25) and can be interpreted as the serum marker of high tumor burden. Qin et al. have found a correlation between tumor burden, measured as the maximum diameter of the primary mass, and the risk of neuroblastoma rupture (9); in the present case series, a statistically significant correlation between tumor diameter and risk of hemorrhage could not be demonstrated.

In the present case series, all the patients who developed hemorrhagic complications presented with severe hypertension (i.e., stage 2), low hemoglobin levels and high serum LDH levels at diagnosis; such features are easy and immediate to detect and can be frequently reassessed with commonly available resources and minimal discomfort for the patient. These three criteria can therefore be used to differentiate patients who have an additional risk of hemorrhage from other high-risk neuroblastoma patients.

All the patients developed hemorrhage after the initiation of chemotherapy. We may speculate that chemotherapy-induced tumor lysis, evidenced by a sharp rise in LDH levels (see Table 2) might cause necrosis of the tissue encasing blood vessels; in such situation, vessel walls that have previously been eroded by neoplastic tissue might be more prone to bleeding. A similar clinical scenario has been demonstrated in patients with metastatic choriocarcinoma and is defined as “choriocarcinoma syndrome” (26). The postulation of a such mechanism in high-risk neuroblastoma patients, however, is speculative; further studies are needed to specifically investigate this issue.

All these patients had systolic pressure above 90th centile for age and height even during active bleeding and under anti-hypertensive treatment; the only abnormality in their hemodynamics was the development of tachycardia (see Table 2). Such observation is in contrast with several studies that report the development of frank hemorrhagic shock secondary to neuroblastoma rupture (7, 9, 13) and highlights the importance of a high index of suspicion and close monitoring of patients who present with the aforementioned risk factors.

Three patients had hemothorax ipsilateral to the primary retroperitoneal tumor. Pleural effusion sometimes can be associated with retroperitoneal neuroblastoma and is generally interpreted as reactive (27); in our cases, we believe that hemothorax can be secondary to the spreading of retroperitoneal hemorrhage through the diaphragmatic crura, as suggested by the angiography performed in patient 2 (Figure 3).

Neuroblastoma patients with hemorrhagic complications need multimodal treatment that should be focused at controlling and limiting the bleeding while supporting vital functions, consisting in blood products administration, crystalloid and colloid infusion, surgical drain of hemothorax and angiographic control of the bleeding source.

Some authors have reported cases of successful emergency surgery on the primary mass in patients with ruptured neuroblastoma (7, 13); in our case series, all the patients with hemorrhagic complications presented with encasement of the aorta and its major branches, that is a well-documented risk factor for major surgical complications and incomplete resection (28–30). In this scenario, emergency surgery on the primary mass should be reserved to patients who do not respond to other therapeutic measures.

The present study has an obvious limitation in its retrospective nature; another limitation is the single-institution design of the study, that reduces the number of patients. The main strong point of this study is the homogeneity of the patients in both groups, who are all affected by high risk neuroblastoma without pre-existing comorbidities, have a comparable prevalence of metastatic disease, a comparable size of the primary tumor and are all treated according to the same protocol; such homogeneity reduces the presence of confounding factors in our analyses.

The result of a perfect separation on multivariate logistic regression is puzzling; it might be the expression of a substantial clinical difference between the two groups, or it might be simply related to the small sample size. Further studies with larger sample size might better clarify such result.

In conclusion, the present data suggest that within the population of patients affected by high-risk neuroblastoma there is a subgroup of children with some specific clinical features, i.e., stage 2 hypertension, anemia, elevated serum LDH levels and multiple vascular IDRF at diagnosis, who have an “additional” risk of developing hemorrhage during induction chemotherapy.

The small sample size of the present study does not allow to establish a clear causal relation; however, we suggest that patients who present with these features at diagnosis are carefully monitored so that hemorrhagic complications are promptly diagnosed and treated before hemorrhagic shock develops.

Further studies are needed to confirm the present observations, define cut-off values for these parameters and design optimal management strategies for these patients.

## Data Availability Statement

The original contributions presented in the study are included in the article/supplementary material, further inquiries can be directed to the corresponding author/s.

## Author Contributions

VV, GP, ACr, ACa, and AI contributed to conception and design of the study. VV and GP organized the database. CM, ASe, and UG revised and analyzed the data. GN and PD revised radiology images. ASt revised the pathology specimens. All authors contributed to manuscript revision, read, and approved the submitted version.

## Conflict of Interest

The authors declare that the research was conducted in the absence of any commercial or financial relationships that could be construed as a potential conflict of interest.

## Publisher's Note

All claims expressed in this article are solely those of the authors and do not necessarily represent those of their affiliated organizations, or those of the publisher, the editors and the reviewers. Any product that may be evaluated in this article, or claim that may be made by its manufacturer, is not guaranteed or endorsed by the publisher.

## References

[1] Yan P Qi F Bian L Xu Y Zhou J Hu J. Comparison of incidence and outcomes of neuroblastoma in children, adolescents, and adults in the United States: a Surveillance, Epidemiology, and End Results (SEER) program population study. Med Sci Monit. (2020) 26:e927218. 10.12659/MSM.927218 33249420 PMC7711874

[2] Irwin MS Park JR. Neuroblastoma: paradigm for precision medicine. Pediatr Clin North Am. (2015) 62:225–562. 10.1016/j.pcl.2014.09.015 25435121

[3] Cohn SL Pearson ADJ London WB Monclair T Ambros PF Brodeur GM. The International Neuroblastoma Risk Group (INRG) classification system: an INRG task force report. J Clin Oncol. (2009) 27:289–97. 10.1200/JCO.2008.16.6785 19047291 PMC2650388

[4] Monclair T Brodeur GM Ambros PF Brisse HJ Cecchetto G Holmes K. The International Neuroblastoma Risk Group (INRG) staging system: an INRG task force report. J Clin Oncol. (2009) 27:298–303. 10.1200/JCO.2008.16.6876 19047290 PMC2650389

[5] Whittle SB Smith V Doherty E Zhao S McCarty S Zage PE. Overview and recent advances in the treatment of neuroblastoma. Expert Rev Anticancer Ther. (2017) 17:369–86. 10.1080/14737140.2017.1285230 28142287

[6] Tolbert VP Matthay KK. Neuroblastoma: clinical and biological approach to risk stratification and treatment. Cell Tissue Res. (2018) 372:195–209. 10.1007/s00441-018-2821-2 29572647 PMC5918153

[7] Meersman A Wojciechowski M Vaneerdeweg W Jorens P Michiels E Ramet J. Acute retroperitoneal hemorrhage and shock as presenting signs of neuroblastoma in an infant. Pediatr Emerg Care. (2008) 24:37–8. 10.1097/pec.0b013e31815f3c60 18212608

[8] Shiokawa N Okamoto Y Kodama Y Nishikawa T Tanabe T Mukai M. Conservative treatment of massive hemothorax in a girl with neuroblastoma. Pediatr Int. (2016) 58:1090–2. 10.1111/ped.13094 27804245

[9] Qin H Yang S Cai S Ren Q Han W Yang W. Clinical characteristics and risk factors of 47 cases with ruptured neuroblastoma in children. BMC Cancer. (2020) 20:243. 10.1186/s12885-020-06720-9 32293329 PMC7092550

[10] High, Risk Neuroblastoma Study 1,.8 of SIOP-Europe (SIOPEN). Available online at: https://clinicaltrials.gov/ct2/show/NCT01704716 (accessed November 18, 2019).

[11] Flynn JT Kaelber DC Baker-Smith CM Blowey D Carroll AE Daniels SR. Subcommittee on screening and management of high blood pressure in children. Clinical practice guideline for screening and management of high blood pressure in children and adolescents. Pediatrics. (2017) 140:e20171904. 10.1542/peds.2017-3035 28827377

[12] Vora D Slovis TL Boal DK. Hemoperitoneum and disseminated intravascular coagulation in two neonates with congenital bilateral neuroblastoma. Pediatr Radiol. (2000) 30:394–7. 10.1007/s002470050769 10876823

[13] Honda S Miyagi H Minato M Kubota KC Okada T. Hemorrhagic shock due to spontaneous rupture of adrenal neuroblastoma in an infant: a rare case and review of the literature. J Pediatr Hematol Oncol. (2012) 34:635–7. 10.1097/MPH.0b013e3182678e1e 23018564

[14] Brock CE Ricketts RR. Hemoperitoneum from spontaneous rupture of neonatal neuroblastoma. Am J Dis Child. (1982) 136:370–1. 10.1001/archpedi.1982.03970400088026 7072672

[15] Normand C Leblond P Mazingue F Nelken B Defachelles AS Bonnevalle M. A case of adrenal haemorrhage after minor trauma in a young child: think of neuroblastoma. Eur J Pediatr Surg. (2006) 16:365–8. 10.1055/s-2006-924605 17160786

[16] Tay M Kapur J. Neuroblastoma with primary pleural involvement: an unusual presentation. Pediatr Radiol. (2011) 41:530–3. 10.1007/s00247-010-1884-3 21079944

[17] Lode HN Henze G Siebert N Ehlert K Barthlen W. Management of tumor rupture and abdominal compartment syndrome in an infant with bilateral high risk stage 4 neuroblastoma: a case report. Medicine. (2019) 98:e16752. 10.1097/MD.0000000000016752 31441848 PMC6716702

[18] Chung C Boterberg T Lucas J Panoff J Valteau-Couanet D Hero B. Neuroblastoma. Pediatr Blood Cancer. (2021) 68:e28473. 10.1002/pbc.28473 33818884 PMC8785544

[19] European Low Intermediate Risk Neuroblastoma Protocol: a SIOPEN Study; Version 5.0; 1st October 2015. Available online at: https://bspho.be/wp-content/uploads/2019/05/LINES-20170102-LINES_Protocol_version-5.0.pdf (accessed July 24, 2021).

[20] Madre C Orbach D Baudouin V Brisse H Bessa F Schleiermacher G. Hypertension in childhood cancer: a frequent complication of certain tumor sites. J Pediatr Hematol Oncol. (2006) 28:659–64. 10.1097/01.mph.0000212995.56812.bb 17023826

[21] Kwok SY Cheng FWT Lo AFC Leung WK Yam MC Li CK. Variants of cardiomyopathy and hypertension in neuroblastoma. J Pediatr Hematol Oncol. (2014) 36:e158–61. 10.1097/MPH.0b013e318290c628 23652880

[22] Strenger V Kerbl R Dornbusch H J Ladenstein R Ambros PF Ambros IM. Diagnostic and prognostic impact of urinary catecholamines in neuroblastoma patients. Pediatr Blood Cancer. (2007) 48:504–9. 10.1002/pbc.20888 16732582

[23] Scaruffi P Morandi F Gallo F Stigliani S Parodi S Moretti S. Bone marrow of neuroblastoma patients shows downregulation of CXCL12 expression and presence of IFN signature. Pediatr Blood Cancer. (2012) 59:44–51. 10.1002/pbc.23339 21994039

[24] Morandi F Barco S Stigliani S Croce M Persico L Lagazio C. Altered erythropoiesis and decreased number of erythrocytes in children with neuroblastoma. Oncotarget. (2017) 8:53194–209. 10.18632/oncotarget.18285 28881804 PMC5581103

[25] Moreno L Guo D Irwin MS Hogarty M Kamijo T Morgenstern D. A nomogram of clinical and biologic factors to predict survival in children newly diagnosed with high-risk neuroblastoma: an International Neuroblastoma Risk Group project. Pediatr Blood Cancer. (2021) 68:e28794. 10.1002/pbc.28794 33205902

[26] Rejlekova K Cursano MC De Giorgi U Mego M. Severe complications in testicular germ cell tumors: the choriocarcinoma syndrome. Front Endocrinol. (2019) 10:218. 10.3389/fendo.2019.00218 31031704 PMC6474390

[27] Gupta H Conrad J Khoury JD McGregor LM Krasin MJ Dome JS. Significance of pleural effusion in neuroblastoma. Pediatr Blood Cancer. (2007) 49:906–8. 10.1002/pbc.21199 17417797

[28] Cecchetto G Mosseri V De Bernardi B Helardot P Monclair T Costa E. Surgical risk factors in primary surgery for localized neuroblastoma: the LNESG1 study of the European International Society of Pediatric Oncology Neuroblastoma Group. J Clin Oncol. (2005) 23:8483–9. 10.1200/JCO.2005.02.4661 16293878

[29] Irtan S Brisse HJ Minard-Colin V Schleiermacher G Galmiche-Rolland L Le Cossec C. Image-defined risk factor assessment of neurogenic tumors after neoadjuvant chemotherapy is useful for predicting intra-operative risk factors and the completeness of resection. Pediatr Blood Cancer. (2015) 62:1543–9. 10.1002/pbc.25511 25820608

[30] Temple WC Vo KT Matthay KK Balliu B Coleman C Michlitsch J. Association of image-defined risk factors with clinical features, histopathology, and outcomes in neuroblastoma. Cancer Med. (2021) 10:2232–41. 10.1002/cam4.3663 33314708 PMC7982630

